# Efficacy and safety of S-1 following gemcitabine with cisplatin for advanced biliary tract cancer

**DOI:** 10.1007/s10637-021-01098-2

**Published:** 2021-04-09

**Authors:** Hiroto Inoue, Akiko Todaka, Kentaro Yamazaki, Kunihiro Fushiki, Hiromichi Shirasu, Takeshi Kawakami, Takahiro Tsushima, Satoshi Hamauchi, Tomoya Yokota, Nozomu Machida, Akira Fukutomi, Yusuke Onozawa, Akira Andoh, Hirofumi Yasui

**Affiliations:** 1 Division of Gastrointestinal Oncology, Shizuoka Cancer Center, 1007 Shimonagakubo, Nagaizumi, Sunto-gun, Shizuoka, 411-8777 Japan; 2 Division of Gastroenterology, Shiga University of Medical Science, Setatsukinowa-tyou, Otsu, Shiga 520-2192 Japan; 3 Division of Medical Oncology, Shizuoka Cancer Center, 1007 Shimonagakubo, Nagaizumi, Sunto-gun, Shizuoka, 411-8777 Japan; 4 Division of Gastroenterology, Kanagawa Cancer Center, 2-3-2 Nakao, Asahi-ku, Yokohama, Kanagawa 241-8515 Japan

**Keywords:** Biliary tract cancer, S-1, Gemcitabine and cisplatin, Second-line therapy

## Abstract

*Background* Combination therapy of gemcitabine with cisplatin (GC) is a standard first-line therapy for unresectable or recurrent biliary tract cancer (BTC). S-1 is often used as a second-line therapy in clinical practice, based on the results of some clinical studies investigating its efficacy and safety following gemcitabine monotherapy. However, few studies have reported on the clinical outcomes of S-1 following GC. The purpose of this study was to elucidate the efficacy and safety of S-1 following GC for unresectable and recurrent BTC. *Methods* We retrospectively collected the data of 116 patients (pts) who were treated with S-1 as a second-line therapy following GC for unresectable or recurrent BTC at Shizuoka Cancer Center (November 2009 to July 2019). *Results* Of these 116 pts., 84 were assessable. Patient characteristics were as follows: intrahepatic bile duct/extrahepatic bile duct/gallbladder cancer, 30/23/31 pts.; metastatic/recurrent/locally advanced, 57/17/10 pts. The median time to treatment failure and overall survival were 2.5 and 6.0 months, respectively. Among 65 pts. with measurable lesions, the overall response rate was 3.1% (2/65 pts) and the disease control rate was 24.6% (19/65 pts). The common grade 3/4 toxicities included anemia (12%), neutropenia (4%), infections (16%), fatigue (6%), and diarrhea (4%). Dose reduction or treatment schedule modification of S-1 was required in 29 pts. (34.5%), and 17 pts. (20%) terminated S-1 due to adverse events. *Conclusions* The efficacy and safety of S-1 following GC were almost the same as those of S-1 following GEM monotherapy for unresectable or recurrent BTC.

## Introduction

The incidence of biliary tract cancer (BTC), including intrahepatic bile duct cancer (IHC), extrahepatic bile duct cancer (EHC), gallbladder cancer (GBC), and ampullary cancer, is increasing worldwide, although this condition is still rare [1]. However, BTC is relatively common in Japan, where it is the sixth leading cause of death [2]. Surgical resection is the only treatment that can provide a cure, but most patients with BTC cannot undergo resection because of metastatic lesions or locally advanced disease at the time of diagnosis. For unresectable and recurrent BTC, systemic chemotherapy is the only effective treatment. The combination chemotherapy of gemcitabine with cisplatin (GC) demonstrated a survival benefit compared with gemcitabine monotherapy in the ABC-02 study [3] and the BT-22 study [4]. Therefore, GC has been recommended as a standard first-line therapy for unresectable and recurrent BTC in several sets of guidelines [5, 6]. In the second-line treatment for BTC, FOLFOX (oxaliplatin, 5-fluorouracil, and leucovorin) significantly improved overall survival compared with active symptom control in a randomized phase III trial (ABC-06) [7], and is described as being a preferred regimen in the National Comprehensive Cancer Network (NCCN) guidelines [6]. However, oxaliplatin is not approved for BTC in Japan. S-1 is an oral fluoropyrimidine anticancer drug that showed a good response rate of 35.0% as first-line therapy for advanced BTC in a phase II study, and was approved in Japan in 2007 [8]. As a second-line treatment for advanced BTC, two phase II studies of S-1 after gemcitabine-based chemotherapy showed response rates of 7.5%–22.7%, with median progression-free survival (PFS) and overall survival (OS) of 2.5–5.4 and 6.8–13.5 months, respectively [9, 10]. Other retrospective studies reported the efficacy of S-1 as a second-line therapy, with response rates of 4%–18.8% and median PFS and OS of 2.3–5.5 and 6.0–8.0 months, respectively [11, 12]. Currently, S-1 is used for patients with advanced BTC for whom GC failed in most Asian countries including Japan. However, most previous studies of S-1 in second-line treatment for advanced BTC were on patients for whom gemcitabine monotherapy failed, and data for patients with a previous history of GC are limited. The purpose of this study was to elucidate the efficacy and safety of S-1 following GC for unresectable and recurrent BTC.

## Patients and methods

### Patients and treatment

We retrospectively assessed the data of 116 patients who were treated with S-1 as second-line therapy following GC for unresectable and recurrent BTC at Shizuoka Cancer Center from November 2009 to July 2019. Unresectable BTC included metastatic BTC and locally advanced BTC defined by invasion of extensive vascular or bile duct, or inadequate remnant liver volume for major hepatic resection. The inclusion criteria were Eastern Clinical Oncology Group (ECOG) performance status (PS) 2 or less and adequate organ function: white blood cell count >3000 μl, neutrophil count >1500/μl, hemoglobin >8.0 g/dl, platelet count >80,000/μl, total bilirubin ≤2.0 mg/dl, serum transaminases <5 times the upper limit, and creatinine clearance ≥30 ml/min. The exclusion criteria were as follows: (1) concomitant use of other anticancer drugs; (2) prior history of S-1; (3) other active malignancies; (4) any other serious complications; and (5) inappropriate initial dose of S-1.

S-1 was administered for 28 days followed by 14 days of rest. Its initial dose was determined based on body surface area (BSA) as follows: BSA < 1.25 m^2^, 80 mg/day; 1.25 m^2^ ≤ BSA ≤ 1.5 m^2^, 100 mg/day; and BSA > 1.5 m^2^, 120 mg/day. When creatinine clearance was ≤60 ml/min, the initial dose of S-1 was reduced by one level (20 mg/day). At the physician’s discretion, the initial dose reduction by one level due to the patient’s general condition, was acceptable. The treatment was repeated until disease progression, appearance of unacceptable adverse events (AEs), or patient refusal, whichever occurred first. Dose reduction and treatment schedule modification of S-1 were considered in the case of severe AEs, such as grade 4 hematological AEs or grade 3 or worse non-hematological AEs according to the Common Terminology Criteria of Adverse Events (CTCAE) version 5.0.

### Evaluations

The outcomes of this study were time to treatment failure (TTF), overall survival (OS), overall response rate (ORR), disease control rate (DCR), and safety. TTF and OS were estimated with the Kaplan–Meier survival curve and compared using the log-rank test. TTF was measured from the date of the first administration of S-1 to the date of discontinuation of S-1 from any cause. OS was measured from the date of the first administration to the date of death from any cause. In addition, as exploratory analysis, the efficacy of the sequential therapy of S-1 following GC was assessed. These efficacy outcomes included TTF2 and OS2, which indicated the periods from the first administration of GC therapy to the discontinuation of S-1 and to death from any cause, respectively.

ORR and DCR were evaluated only in the patients with measurable lesions. ORR was the ratio of the number of patients achieving a complete or partial response to the total number of patients with measurable lesions, and DCR was the ratio of the number of patients achieving a complete or partial response, or stable disease to the total number of patients with measurable lesions, in accordance with the Response Evaluation Criteria in Solid Tumors (RECIST) version 1.1. All statistical analyses were performed using EZR version 1.37 (Saitama Medical Center, Jichi Medical University, Saitama, Japan) [13], which is a graphical user interface for R (The R Foundation for Statistical Computing, Vienna, Austria).

## Results

### Patient characteristics

**Fig. 1:**
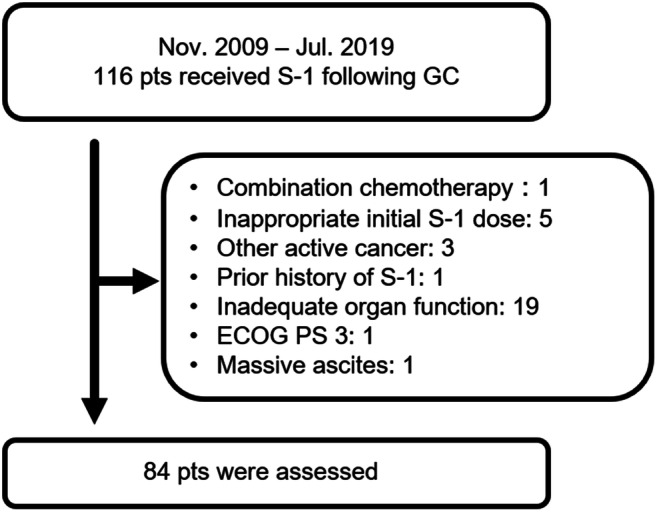
Flow diagram of the patients included in this study

Among 116 patients who were treated with S-1 as second-line therapy following GC for unresectable or recurrent BTC, 32 patients were excluded in this analysis: 19 patients had inadequate organ function, 5 patients received S-1 with an initial dose reduction of two levels or an inappropriate dose, and 3 patients had other active cancer (Fig. 1). Thus, the analysis set in this study included 84 patients; a summary of the patients’ characteristics is shown in Table 1. There were 30 (36%), 23 (27%), and 31 (37%) patients with IHC, EHC, and GBC, respectively. There were no patients with ampullary cancer. There were 57 (68%), 17 (20%), and 10 (12%) patients with metastatic, recurrent, and locally advanced disease, respectively.

**Table 1:** Characteristics of all patients

N		84
Age (years)	Median (range)	66 (29–79)
Sex	Male	52 (62%)
	Female	32 (38%)
ECOG PS*	0	40 (48%)
	1	33 (39%)
	2	11 (13%)
Primary lesion	Intrahepatic	30 (36%)
	Extrahepatic	23 (27%)
	Gallbladder	31 (37%)
Disease status	Metastatic	57 (68%)
	Recurrent	17 (20%)
	Locally advanced	10 (12%)

*Eastern Cooperative Oncology Group performance status

About GC treatment, 2 patients discontinued GC for adverse events: renal failure and interstitial lung disease. In 17 patients, cisplatin was interrupted due to renal failure, peripheral neuropathy, and cumulative dose of CDDP. After that, only gemcitabine was continued until disease progression.

### Treatment

At the data cut-off date of this analysis of January 2020, all patients terminated S-1 and median follow-up time was 5.7 months. Initial dose reduction was required in 13 patients (16%). The reasons for initial dose reduction were as follows: poor general condition in 9 patients; older age in 3 patients; and thrombocytopenia in 1 patient. Furthermore, dose reduction or treatment schedule modification after the initiation of S-1 was required in 29 patients (34.5%). The reasons for this were as follows: fatigue or anorexia in 12 patients; diarrhea in 6 patients; and creatinine increase in 3 patients. Sixty-four patients (76%) terminated S-1 due to disease progression, while 17 patients (20%) terminated it due to AEs: infection in 7 patients, and fatigue or anorexia in 4 patients. Thirteen patients (15%) received subsequent anticancer treatments after the discontinuation of S-1 and eight patients received gemcitabine-based regimens.

### Efficacy

**Fig. 2:**
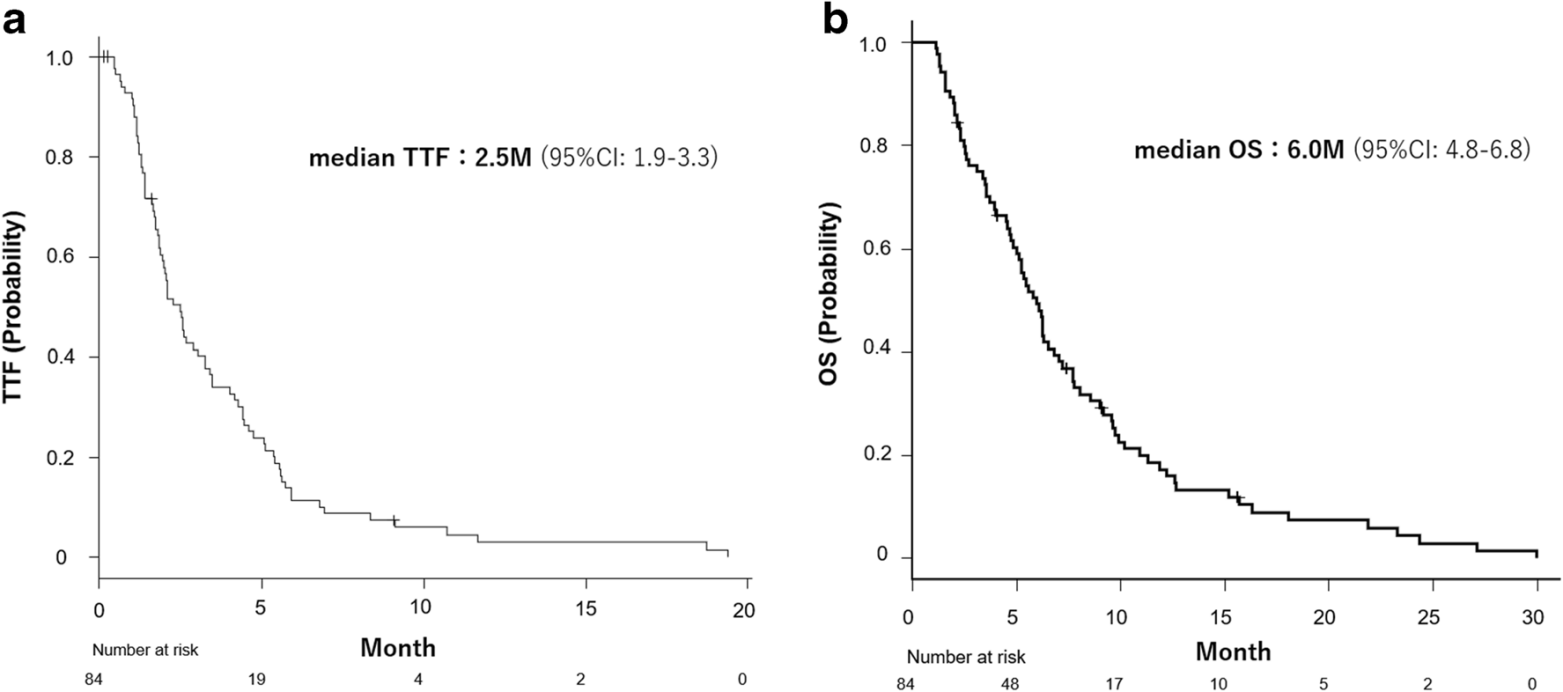
Kaplan–Meier plots of (**a**) time to treatment failure and (**b**) overall survival

The median TTF and OS were 2.5 (95% CI: 1.9–3.3) and 6.0 months (95% CI: 4.8–6.8), respectively (Fig. 2). Among 65 patients with measurable lesions, no patients achieved complete response, and partial response and stable disease were observed in 2 (3.1%) and 14 (21.5%) patients, respectively, resulting in ORR of 3.1% and DCR of 24.6% (Table 2).

**Table 2:** Overall response of the patients with measurable lesions

	*N* = 65
Complete response	0
Partial response	2 (3.1%)
Stable disease	14 (21.5%)
Progressive disease	45 (69.2%)
Not evaluable	4 (6.2%)
Overall response rate	3.1%
Disease control rate	24.6%

**Fig. 3:**
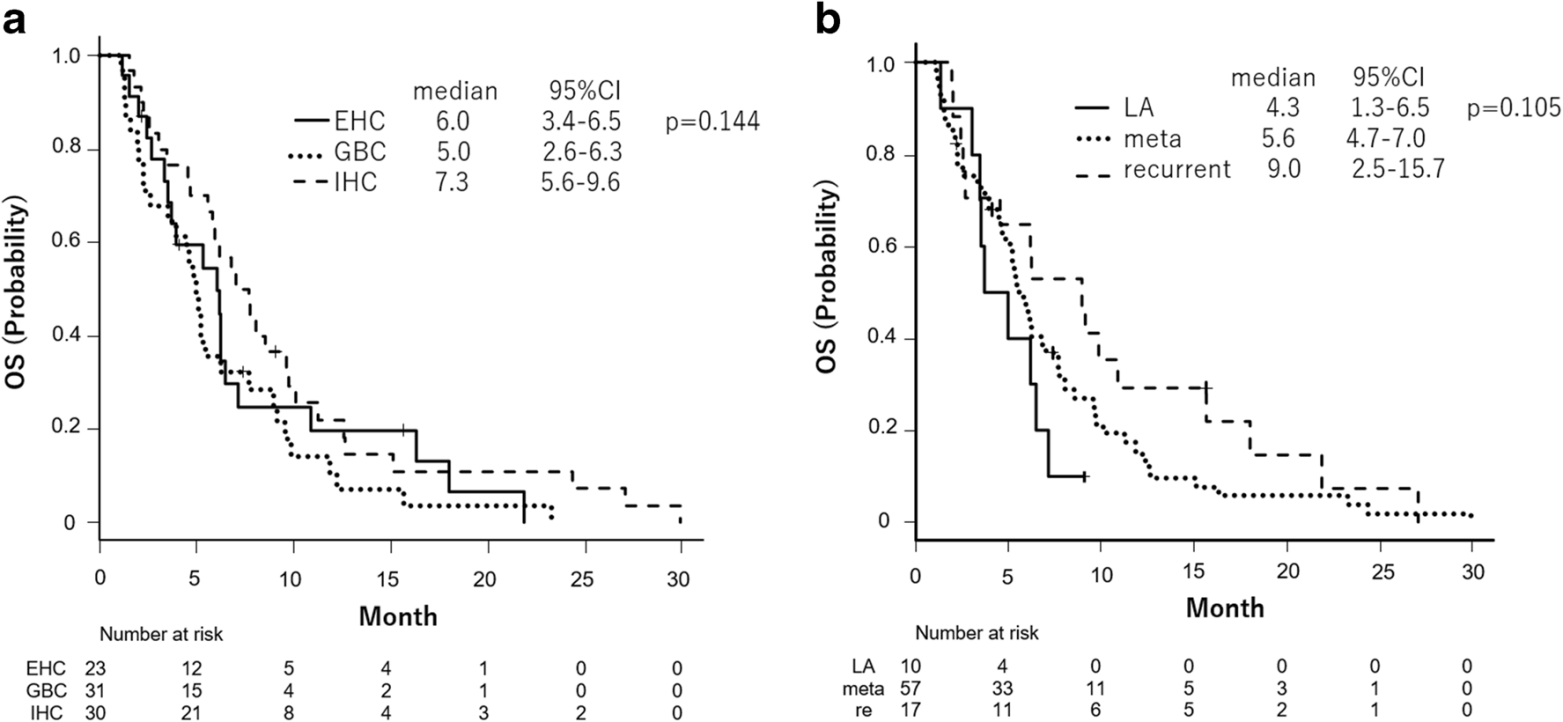
Kaplan–Meier plots of overall survival (**a**) stratified by primary lesion and (**b**) disease status

In the subgroup analysis by primary lesion, the median OS of the patients with IHC, EHC, and GBC was 7.3 (95% CI: 5.6–9.6), 6.0 (95% CI: 3.4–6.5), and 5.0 months (95% CI: 2.6–6.3), respectively, which did not differ significantly (*p* = 0.144). ORR and DCR of the patients with each primary lesion were as follows: 4% (1/25) and 32% (8/25) in IHC patients; 0% and 14.3% (2/14) in EHC patients; and 3.8% (1/26) and 23.1% (6/26) in GBC patients, respectively. In the analysis by disease status, the median OS of the patients with metastatic, recurrent, and locally advanced disease was 5.6 (95% CI: 4.7–7.0), 9.0 (95% CI: 2.5–15.7), and 4.3 months (95% CI: 1.3–6.5), respectively, which did not differ significantly (*p* = 0.105) (Fig. 3).

As exploratory analysis, the median TTF2 and OS2 were 11.2 (95% CI: 8.0–13.1) and 14.6 months (95% CI: 11.8–17.1), respectively. The median period of prior GC treatment was 6.0 months (95% CI: 4.4–7.2). Except for 19 patients who discontinued cisplatin, the median TTF and OS of S-1 were 2.1 (95% CI: 1.7–3.3) and 5.1 months (95% CI: 3.7–7.0), respectively.

### Safety

The major AEs of grade 2 or higher are shown in Table 3. The major grade 3 or 4 hematological toxicities included anemia (12%) and neutropenia (4%). The common grade 3 or 4 non-hematological toxicities included infections (16%), fatigue (6%), and diarrhea (4%). Of 14 patients with grade 3 or 4 infections, 7 patients had biliary tract infections. No treatment-related death was observed.

**Table 3:** Adverse events (grade 2 or above)

	Grade 2 (%)	Grade 3/4 (%)
Leukopenia	2 (2%)	1 (1%)
Neutropenia	1 (1%)	3 (4%)
Anemia	7 (8%)	10 (12%)
Thrombocytopenia	12 (14%)	1 (1%)
Febrile neutropenia	0	2 (2%)
Nausea	11 (13%)	0
Anorexia	19 (23%)	2 (2%)
Fatigue	19 (23%)	5 (6%)
Diarrhea	8 (10%)	3 (4%)
Abdominal pain	0	2 (2%)
Mucositis oral	2 (2%)	0
Watering eyes	2 (2%)	0
Skin rash	1 (1%)	0
Creatinine increase	5 (6%)	0
Colonic perforation	0	1 (1%)
Biliary tract infection	0	7 (8%)
Infection (others)	5 (6%)	7 (8%)

## Discussion

To the best of our knowledge, this is the first study assessing the efficacy and safety of S-1 for patients with unresectable and recurrent BTC for whom GC, the current standard first-line therapy, failed. Our results regarding the efficacy were similar to those of some previous studies after gemcitabine monotherapy refractory: Suzuki et al. [9] reported median PFS and OS were 2.5 and 6.8 months, respectively, and Kobayashi et al. [11] reported median PFS and OS were 2.3 and 6.0 months, respectively. S-1 has a certain efficacy for patients who have become refractory to not only gemcitabine monotherapy but also GC combination therapy. Among the previous studies, however, that by Sasaki et al. [10] showed better results than other studies: median PFS and OS were 5.4 and 13.5 months, respectively. The reason for the difference between our work and this previous report may be related to the rate of primary lesions and disease status. Our study included 37% GBC cases and 20% cases with recurrent disease, while that of Sasaki et al. included a smaller proportion of GBC and a larger proportion of recurrent disease. Patients with GBC have been reported to have a shorter OS than those with IHC/EHC [14, 15]. Okusaka et al. [4] reported that patients with primary tumors had worse survival than those without primary tumors. This means that recurrent disease after primary tumor resection is likely to show better survival benefits. These tendencies were also found in our study, although there was no significant difference.

Because of second-line therapy, ORR in this study (3.1%) was not so good compared to ORR as first-line therapy (35.0%) [8] This result was similar to the previous study as second-line therapy (4.0–7.5%) Therefore, it is considered difficult to improve the symptoms caused from the tumor.

The efficacy of the sequential therapy of S-1 following GC appears to be relatively good: the median TTF2 and OS2 were 11.2 and 14.6 months, respectively. Because the median period of GC treatment was consistent with those of previous studies [3, 4], the second-line therapy of S-1 benefited survival. The triplet regimen of GC plus S-1 (GCS) became one of the standard therapies in Japan, based on a phase III study (KHBO1401-MITSUBA) in 2018 [16]. GCS showed significantly longer PFS (7.4 vs. 5.5 months) and OS (13.5 vs. 12.6 months), and higher ORR (41.5% vs. 15.0%) than GC; however, the effect of adding S-1 to GC on OS was less than 1 month. Of the patients who received GC in the KHBO1401 study, only 22.8% were treated with S-1 after becoming refractory to GC. In the JCOG1113 study, 53% of patients in the GC group were treated with S-1 in second-line therapy, and the median OS of the patients receiving GC was 13.4 months [17]. It should be noted that the results were limited to cases that could be treated with S-1 after becoming refractory to GC, although the OS of sequential therapy in both JCOG1113 and our study was comparable to that of the triplet regimen. If the triplet therapy is difficult, sequential therapy is also expected to be a good option. It is important to establish an appropriate strategy that exploits all three drugs: gemcitabine, cisplatin, and S-1.

As with the efficacy, our results about safety were also almost consistent with those of previous reports, except for increases in anemia and creatinine. In an effort to make this cohort as close as possible to real-world patients, our inclusion criteria were made slightly wider than in previous studies, within an acceptable range. For that reason, the rate of grade 3/4 anemia (12%) was slightly higher than those of previous reports (2%–8%) [9, 10, 12]. Similarly, the rate of grade 2 creatinine increase was also higher. Nevertheless, few patients terminated S-1 treatment due to hematological toxic effects and renal dysfunction. Many patients terminated S-1 due to infection including biliary tract infection, which was mainly caused by the tumor characteristics rather than drug toxicity. Thus, S-1 following GC appears to be a feasible treatment, with appropriate interruption and dose or schedule modification.

Our study has several limitations. First, this is a retrospective study at a single institution. Even so, our study is the largest to assess the effect of S-1 for second-line treatment of unresectable and recurrent BTC, and the first study limited to patients for whom GC failed. Second, this is not a comparative study: we did not compare the results of S-1 following GC with those of other anticancer drugs or placebo following GC. Although S-1 has been mainly used only in Asian countries, S-1 is the only currently available drug for second-line treatment of unresectable and recurrent BTC. However, the FOLFOX regimen may become available as a second therapy in the near future, and target therapies based on genomic alterations are also likely to become effective treatments [18]. These new therapies may reduce the opportunity to use S-1 for second-line therapy of unresectable and recurrent BTC. Nonetheless, not all patients are indicated for these new therapies, so the current study is meaningful in clinical practice. S-1 is still a treatment option with the advantages of less toxic effects and the convenience associated with oral drug administration.

In conclusion, the efficacy and safety of S-1 following GC were almost the same as those of S-1 following GEM monotherapy. S-1 is a feasible treatment and has a certain effect, although its efficacy is marginal. Nonetheless, the sequential therapy of S-1 following GC showed favorable effects. Therefore, when starting treatment with GC, it is necessary to make an appropriate strategy for using S-1 sequentially, and to exploit all three drugs.

## Data Availability

All data generated or analysed during this study are included in this published article.
